# Current Progress of Research on Neurodegenerative Diseases of Salvianolic Acid B

**DOI:** 10.1155/2019/3281260

**Published:** 2019-06-24

**Authors:** Rui Zhao, Xifang Liu, Lixin Zhang, Hao Yang, Qian Zhang

**Affiliations:** ^1^Translational Medicine Center, Hong Hui Hospital, Xi'an Jiaotong University, Xi'an 710054, China; ^2^College of Pharmacy, Shaanxi University of Chinese Medicine, Xi'an 712046, China; ^3^Department of Chinese Medicine Orthopaedic, Hong Hui Hospital, Xi'an Jiaotong University, Xi'an 710054, China; ^4^Department of Pharmacy, Hong Hui Hospital, Xi'an Jiaotong University, Xi'an 710054, China

## Abstract

*Salvia miltiorrhiza* Bunge (Lamiaceae), one of the most commonly used traditional Chinese herbs, is widely used for the treatment of cardiovascular disease, cerebrovascular disease, Alzheimer's disease, and Parkinson's disease in clinical practice. Salvianolic acid B (Sal B, C_36_H_30_O_16_, FW = 718.62) is the main water-soluble active ingredient of *Salvia miltiorrhiza* Bunge, which performs prophylactic and therapeutic activities against neurodegenerative diseases. So far, numerous studies have proved that multiple factors and mechanisms are involved in the pathological process of neurodegenerative diseases, including amyloid *β* (A*β*) aggregation and fibril formation, hyperphosphorylation of tau protein, neuroinflammation, oxidative-stress damage, mitochondrial dysfunction, and neuron apoptosis. This study is aimed at reviewing experimental studies and describing the possible mechanisms of Sal B on neurodegenerative diseases.

## 1. Introduction

Salvianolic acid B (Sal B) is the main water-soluble active ingredient of *Salvia miltiorrhiza* Bunge (Lamiaceae), which is one of the most commonly used traditional Chinese herbs. *Salvia miltiorrhiza* Bunge is widely distributed in both China and Japan and has been widely accepted as a health product in western countries in recent years, owing to its remarkable and reliable biological effects. In clinical practice, it is used for the treatment of various diseases, including cardiovascular disease, cerebrovascular disease, Alzheimer's disease, Parkinson's disease, and renal injury [1–4].

**Figure 1:**
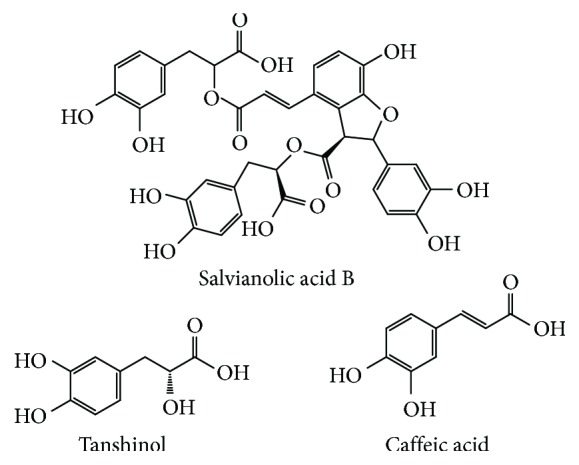
Chemical structures of salvianolic acid B, tanshinol, and caffeic acid.

As the highest content in *Salvia miltiorrhiza* Bunge, Sal B accounts for 3%-5% of the total dry weight of the herb. Meanwhile, Sal B possesses a variety of pharmacological activities, including antagonizing atherosclerosis, alleviating myocardial ischemia/reperfusion injury, and protecting the nerve system [5, 6]. Because of the most abundant content and good pharmacological activity, Sal B is regarded as a landmark ingredient for the identification of *Salvia miltiorrhiza* Bunge. Chinese pharmacopoeia (2015 Edition) also explicitly takes it as an index competent for content determination and stipulates that the content should not be less than 3% [7]. The pure product of Sal B is a white powder with poor thermal stability, which is hygroscopic and soluble in water, methanol, and ethanol. Sal B is formed by the condensation of 3 molecules of tanshinol and 1 molecule of caffeic acid (structure is shown in Figure 1). In the process of decoction and concentration, a small amount of it hydrolyzes to produce alkannic acid and tanshinol, and a part of tanshinol could further transform into rosemary acid under acidic conditions [8]. More importantly, there are nine phenolic hydroxyls that exist in Sal B, which could provide H^+^ so as to prevent the lipid peroxidation of cells. So far, plenty of studies have shown that Sal B possesses a satisfactory free-radical scavenging effect, and its antioxidant activity is even stronger than glutathione, vitamin E, and Ginkgo biloba extract [4]. Besides, as a promising molecule, Sal B possesses low toxicity (LD_50_ = 636.89 mg/kg, mice, tail vein injection) [9] and could cross the blood-brain barrier [10]. Thus, it has the advantage of being developed into a potential drug for the central nervous system (CNS).

Neurodegenerative diseases are a heterogeneous group of disorders characterized by gradually progressive, selective loss of anatomically or physiologically related neuronal systems. It can be broadly classified by their clinical presentations, with extrapyramidal and pyramidal movement disorders and cognitive or behavioral disorders being the most common [11]. By and large, they can be divided into acute neurodegenerative diseases and chronic neurodegenerative diseases. The former mainly includes cerebral ischemia (CI), brain injury (BI), and epilepsy. And the latter includes Alzheimer's disease (AD), Parkinson's disease (PD), Huntington's disease (HD), amyotrophic lateral sclerosis (ALS), spinal cerebellar ataxia (SCA), and Pick's disease [12]. Although different neurodegenerative diseases are typically defined by specific protein accumulations and anatomic vulnerability, neurodegenerative diseases share many fundamental processes associated with progressive neuronal dysfunction and death, such as proteotoxic stress and its attendant abnormalities in ubiquitin-proteasomal and autophagosomal/lysosomal systems, oxidative stress, programmed cell death, and neuroinflammation [13–15].

In recent years, a large number of studies have shown that Sal B also has a good prospect in prevention and treatment of neurodegenerative diseases. This paper summarizes the role of Sal B in this field and provides references for further research and application.

## 2. Effects of Sal B on *β*-Amyloid

The principal component of senile plaques in AD is a 42 aa peptide, referred to as *β*-amyloid (A*β*). A*β* is generated from a family of differentially spliced, type-1 transmembrane domain-containing proteins, called amyloid precursor protein (APP), by endoproteolytic processing [16]. Under physiological conditions, the majority of APP is cleaved by *α*-secretase (the extracellular region) into a fragment of 83 amino acids (C83) and an extracellular domain (sAPP*α*). sAPP*α* is further cleaved by *γ*-secretase. The cleavage site of *α*-secretase can preclude A*β* formation and deposition, which is beneficial for the neurons' protection and cell proliferation. An alternate secretory pathway, enriched in neurons and brain, leads to the cleavage of APP at the N terminus of the A*β* peptide by *β*-secretase, thus generating a C-terminal membrane-bound fragment (C99) and an extracellular domain (sAPP*β*). This fragment (between residues 38 and 43) is subsequently cleaved by *γ*-secretase(s), generating both A*β*_40_ (90%) and A*β*_42_ (10%) [17, 18]. In an aqueous solution, A*β* exists in a mixed form as an *α*-helix, free-curling, and *β*-folding structure. Thereupon, the *β*-folding structure tends to gather into polymers and then forms A*β* fibers and neurotoxic plaques of aging. A mutation of the APP gene and an imbalance of the A*β* removal mechanism cause the excessive production of A*β*, while the neurotoxicity of A*β* itself amplifies the response of the CNS to various injury stimulations, which eventually interferes with the normal function of neurons and causes cell necrosis or apoptosis, leading to dementia [19]. Therefore, the deposition of A*β* has been recognized as the main pathogenesis of AD.

### 2.1. Inhibition of A*β* Aggregation and Fiber Formation

In in vitro cell experiments, Sal B has been confirmed as an effective inhibitor of A*β* aggregation and fibrogenesis. Sal B was incubated with A*β*_40_ (100 mg/L) at 25°C for 30 h, 48 h, and 100 h, and the results showed that Sal B (10 nmol/L) could significantly inhibit fiber formation of A*β* in PC12 [20]. In the studies of Durairajan et al. [21], fluorescence spectroscopic analysis with thioflavin T and A*β* concentration immunoassay also confirmed that Sal B could dose-dependently (1-100 *μ*M) and time-dependently (3-7 d) inhibit A*β*_40_ fiber formation (IC_50_: 1.54-5.37 *μ*M), as well as damage the formed A*β*_40_ fiber (IC_50_: 5.00-5.19 *μ*M). In addition, the pretreatment of Sal B could reduce the accumulation of reactive oxygen induced by A*β*_25-35_ aggregation in PC12 cells. The results of electron microscopy and circular dichroism spectrum also showed that Sal B pretreatment could inhibit the aggregation and formation of A*β*_40_ fiber at various stages in a dose-dependent and time-dependent manner. In the study of Tang et al., Sal B (25 *μ*M, 50 *μ*M, and 100 *μ*M) significantly reduced the generation of A*β*_40_ and A*β*_42_ in culture media by decreasing the protein expressions of sAPP*β* and *β*-site APP-cleaving enzyme 1 (BACE1); BACE1 is a type I integral membrane protein belonging to the pepsin family of aspartyl proteases and acts as the *β*-secretase in SH-SY5Y-APPsw cells. Meanwhile, Sal B increases the levels of sAPP*α* and antidisintegrin and metalloprotease 10 (ADAM10); ADAM10 possesses *α*-secretase activity and belongs to the ADAM family (a disintegrin and metalloproteinase family enzyme) [22]. As a natural antioxidant possessing neuroprotective effects, Sal B performs better than ferulic acid but slightly poorer than curcumin as an A*β* aggregation inhibitor [20, 21].

The mechanisms by which Sal B inhibits the formation and aggregation of A*β* fibers are as follows: (1) as polyphenols, Sal B possesses good oxygen free-radical scavenging effects; (2) the structure of the phenolic hydroxyl group could effectively inhibit the generation of new oxygen free radicals; and (3) Sal B molecules contain a di-3,4-phenol hydroxyl structure, which is the same as that in rosmarinic acid. This structure has been proven to prevent A*β* aggregation by binding to free A*β* [23].

### 2.2. Inhibition of Cytotoxicity Induced by A*β*

The results of methyl thiazolyl tetrazolium (MTT) showed that A*β*_40_ has significant toxic effects on PC12 cells after incubation for 30 h, whereas this toxic effect could be significantly reduced by Sal B (10 nmol/L, 100 nmol/L). Meanwhile, Sal B also exhibits good protective effects on PC12 cells which were incubated with aged A*β*_25-35_ for 48 h [24]. Feng and Zhang established the injury model on primary rat cortical neurons using 5 *μ*mol/L A*β*_40_. Then, 0.01 *μ*g/L, 0.10 *μ*g/L, and 1.00 *μ*g/L of Sal B were added and incubated for 24 h. The results of the MTT and Griess experiment showed that Sal B could significantly increase cell viability and reduce lactate dehydrogenase (LDH) release rate and NO release in a dose-dependent manner, thereby alleviating the toxicity of A*β*_40_ on cortical neurons [25].

## 3. Effect of Sal B on Tau Protein Hyperphosphorylation

Tau protein is a kind of highly soluble microtubule-associated cytoskeleton protein. It can interact with tubulin to stabilize microtubules and promote tubulin assembly into microtubules, which then combine with neurofilaments to form neurofibrillary tangles. However, the abnormal hyperphosphorylation of tau protein could cause the structural destruction and functional disorder of microtubules, which assemble in the form of paired helical filaments (PHFs). Furthermore, abnormal saccharification/glycosylation of PHFs causes nerve fiber tangles (neurofibrillary tangles, NFTs) in nerve cells and then induce the loss of cell function. NFTs could induce a wide cross-connection between different molecules and influence the information transfer between cells. Studies have shown that the number of NFTs is correlated with the severity of AD, and the hyperphosphorylation level of tau protein in the brain of AD patients is abnormally high [26]. Therefore, in addition to the deposition of A*β*, the hyperphosphorylation of tau protein and the degeneration of nerve fibers are major pathological features of AD and comprise its pathogenesis.

The proteases that promote the phosphorylation of tau protein are mainly glycogen synthase kinase-3 (GSK-3), cyclin-dependent kinase 5 (CDK-5), and mitogen-activated protein kinase (MAPK). Studies have confirmed that A*β* causes the phosphorylation of tau protein, destabilizes microtubules, and leads to neuron death. Therefore, the aggregation of A*β* and the formation of fibrils may serve as the initiation link, while the dysfunction of tau protein plays a key role in the process of neuron dysfunction and death [27].

Okadaic acid (OA) is used to establish cell and animal models of tau protein phosphorylation because of its selective activation of GSK-3*β*. In the study of Tang et al. [28], the MTT assay showed that the cell activity of SH-SY5Y cells was reduced to 62.3% after being incubated with 50 nmol/L OA for 36 h, compared with normal cells. However, after preincubation with Sal B, the decrease in SH-SY5Y cell activity caused by OA is significantly alleviated (0.072 *μ*g/mL: *P* < 0.05; 0.72 *μ*g/mL: *P* < 0.01). In the Hoechst 33258 staining and membrane protein V-FITC/PI dual-labeling experiments, the cell apoptosis rate is 34.2% after treatment with 50 nmol/L OA. However, after treatment with 0.072 *μ*g/mL OA, 0.72 *μ*g/mL in advance, the cell morphology gradually returns to normal, the number of nuclear condensation gradually declines, and the rate of cell apoptosis decreases to 13.7% and 20%, respectively. All the above results show that Sal B could alleviate the cell damage caused by the hyperphosphorylation of tau protein. In another research, Tang et al. found that the phosphorylation at Ser9 of GSK-3*β* was significantly increased after the exposure of SalB for 24 h (50 *μ*M, 100 *μ*M) in SH-SY5Y-APPsw cells. And this effect might be related to the suppression of BACE1 expression and amyloidogenesis [22].

## 4. Anti-Inflammatory and Antioxidant Effects of Sal B

Many studies suggested that nerve inflammation and oxidative damage play an important role in the onset of AD. The innate immune system, as a double-edged sword, is formed in the process of biological evolution and could react quickly to various harmful substances to protect the body. Long-term and uncontrolled stimulation of harmful substances, such as aggregated A*β*, activates the innate immune system, which causes damage to the brain. The major characteristic of brain inflammation is the activation of glial cells, especially microglia. Various factors induce the release of preinflammatory cytokines and oxygen free radicals of microglia cells, which initiate or amplify the neuron damage. Then, the damaged neurons in turn activate more microglia cells, forming a vicious cycle and aggravating the disease process [29, 30]. For example, McGeer and McGeer's study showed that abundant activated microglia cells exist in the brain of AD patients. These activated microglia cells could secrete neurotoxic substances, such as complement protein and inflammatory factors, so as to activate immune inflammatory reactions and generate neurotoxicity [31]. At present, the relationship between the activation of glial cells and nerve injury has not been clearly elucidated, but glial cells have become a research hotspot and therapeutic target for neurodegenerative diseases [32].

In the study of Lee et al. [33], A*β*_25-35_ was administered to rats by intraventricular injection, and Sal B (10 mg/kg) was administered 1 h later, a procedure which was conducted for 7 successive days. Immunohistochemical results showed that the number of microglia (OX-42 positive) and astrocytes (GFAP positive), originally elevated by the activation of A*β*_25-35_, significantly decreased because of the effect of Sal B in the CA1, CA3, and DG regions of the hippocampus. Meanwhile, mRNA expression of inducible nitric oxide synthase (iNOS) and cyclooxygenase (COX-2) that originally increased also significantly decreased due to the Sal B effect. Similar results were also observed in the study of Chen et al. [34]. After building a mice model of traumatic brain injury, 25 mg/kg Sal B was administered for intervention. Immunohistochemical and enzyme-linked immunosorbent assay (ELISA) results showed that expressions of proinflammatory cytokines tumor necrosis factor-*α* (TNF-*α*) and interleukin-1*β* (IL-1*β*) were significantly reduced and expressions of anti-inflammatory factors IL-10 and TGF-*β*1 were significantly increased. In the study of He et al. [35], the primary neurons of C57BL/6J mice were exposed to 500 nM A*β* to establish the AD model, then Sal B (50-400 *μ*M) was incubated for 28 h which substantially alleviated intraneuronal glutathione (GSH) and lipid oxidation.

Wang et al. investigated the effect of Sal B on microglial activation in a microglia-neuron coculture system. In terms of anti-inflammatory effects, microglia cells induced by 1 *μ*g/mL lipopolysaccharide (LPS) could produce mass NO, TNF-*α*, and IL-1*β*. After incubation with 50 *μ*M Sal B for 24 h, ELISA and Griess tests showed that the production of NO, TNF-*α*, and IL-1*β* was significantly decreased (*P* < 0.01); meanwhile, PRC results showed that the mRNA expressions of iNOS, TNF-*α*, and IL-1*β* were also significantly decreased (*P* < 0.05). In terms of antioxidant effects, the amount of production of reaction oxygen species (ROS) in microglia cells induced by LPS could be significantly reduced by Sal B (0.5 *μ*M, 5 *μ*M, and 50 *μ*M) in a dose-dependent manner (*P* < 0.01). Further investigation indicated that these effects of Sal B mentioned here were conducted by inhibiting the activation of transcription factor NF-*κ*B in microglia. These data demonstrate that Sal B plays a positive role for preventing microglia-mediated neuroinflammation [36].

## 5. Effect of Sal B in Inhibiting Apoptosis

Apoptosis involves the activation, regulation, and expression of a series of genes. There are two classical apoptosis pathways: the extrinsic or death receptor pathway and the intrinsic or mitochondrial pathway. In the former pathway, death receptor activation, such as CD95/APO-1/Fas and TNF receptor 1 (TNFR1), is initiated by specific ligands called death activators, such as APO-1/Fas, TNF-*α*, and TNF-related apoptosis inducing ligand (TRAIL). Then, the death domain of the intracellular segment of the death receptor attracts adapter proteins, which then recruit procaspase-8, leading to its activation. Activated caspase-8 can then activate other initiator and executioner caspases, either directly or indirectly, by cleaving Bid. In the latter pathway, many stimuli influence the inner mitochondrial membrane to release proapoptotic proteins (such as bax and bak) from the intermembrane space into the cytosol. Then, mitochondria is induced to release cytochrome *c* (Cyt *c*), apoptosis-inducing factor (AIF), and other promoting polypeptides to enter the cytoplasm, which mediates the activation of the downstream caspase and induces apoptosis [37]. Evidences indicated that mitochondria involvement is also required in receptor-mediated apoptosis pathways. Meanwhile, these two pathways are linked and molecules in one pathway can influence another [38].

Evidence from animal models and tissue cultures support the implication of apoptosis in mechanisms leading to neurodegenerative diseases. In autoptic brains from neurodegenerative disease patients, many dying neurons have been identified, most of them showing morphological features of apoptosis and an increase in the expression levels of proapoptotic factors. Many signals can initiate apoptosis in neurons, including caspase, bax, bad, bcl-2, bcl-XL, glutamate receptor proteins, Fas, Par-4, p53, telomerase, protein kinase C, protein kinase C, and calcium-binding proteins [39].

Studies have indicated that cognitive dysfunction in AD patients is closely related to neuron apoptosis in the CNS. Moreover, the apoptosis rate in the brain of AD patients is 50 times faster than that in the normal control group [40]. In many types of cells, A*β* aggregation and fibrogenesis have been shown to be neurotoxic and play a key role in the pathogenesis of AD, leading directly to neuron apoptosis. In the studies of Liu et al. and Tang and Zhang, flow cytometry was used to detect the effect of Sal B on the apoptosis of PC12 cells induced by A*β*_25-35_. Results showed that PC12 cells treated with A*β*_25-35_ exhibited degraded DNA content characteristic of apoptosis cells (19.9%). However, PC12 cells pretreated with Sal B (10 nmol/L, 100 nmol/L, and 1 *μ*mol/L) manifested a relatively low proportion of apoptosis (15.7%, 13.5%, and 11.8%, respectively). In addition, in a study using a cell-free apoptotic system, no protective effect of Sal B against apoptotic cytoplasmic extracts induced nuclei cleavage. The result may indicate that Sal B protects the cells at a site more upstream than these apoptotic effectors [20, 41].

Currently, 1-methyl-4-phenylpyridine (MPP+) is the most commonly used reagent to construct animal and cell models for researching the apoptosis mechanism in PD. It can be absorbed into dopaminergic neurons, it can selectively inhibit the mitochondrial electron transport chain (ETC), it can open the mitochondrial permeability transition hole (MPTP), and it can cause mitochondrial membrane potential to collapse and release Cyt *c*, thus leading to the apoptosis of dopaminergic neurons [42]. In the study of Zeng et al., all the characteristics mentioned above were observed in SH-SY5Y cells exposed to MPP+ (500 *μ*Μ) for 24 h. After pretreatment with Sal B (50 *μ*M, 100 *μ*M) for 2 h, the results of MTT and flow cytometry showed a stabilization of mitochondrial membrane potential and a decrease of apoptotic cell number and ROS production. Western blot (WB) results showed that Sal B inhibited the release of Cyt *c* and the activation of caspase-3 (*P* < 0.01). Polymerase chain reaction (PCR) results showed that pretreatment with Sal B could significantly reduce the bax expression to a normal level, thereby reducing the increased ratio of bax/bcl-2 due to exposure to MPP+ [43]. Similar results were observed in the research of Tian et al. PD models of SH-SY5Y were established by exposure to 6-hydroxydopamine (100 *μ*M) for 12 h. Pretreatment with Sal B (0.1 *μ*M, 1 *μ*M, and 10 *μ*M) could dose-dependently reduce the activity of caspase-3 and inhibit the increase in bax and the decrease of bcl-2, thereby increasing the proportion of bcl-2/bax reduced by exposure to 6-hydroxydopamine (6-OHDA) [44].

## 6. Effect of Sal B on Mitochondrial Dysfunction

The series of events that lead to neurodegeneration are intricate. Various neurodegenerative disorders manifest with different symptoms and affect different parts of the brain. Mitochondrial dysfunctions are considered as conjunctive features—a point of convergence to different pathological pathways. Mitochondria play an important role in cell processes, signaling pathways, calcium homeostasis, cell cycle regulation, apoptosis, ROS production, and thermogenesis. Mitochondrial dysfunction, increased ROS production, and oxidative damage are responsible for numerous neurodegenerative disorders. Apoptosis and excitotoxicity are the two significant grounds of neuronal cell death, and the role of mitochondria is crucial in both cases. Increased ROS production in the neurodegenerative process might affect mitochondrial parameters as well as adenosine triphosphate (ATP) production, membrane potential, MPTP activation, and calcium uptake. These changes can lead to and result in neuronal damage [45, 46].

More recently, evidence for impaired mitochondrial dynamics (shape, size, fission-fusion, distribution, movement, etc.) in neurodegenerative diseases has emerged. The first evidence of the involvement of mitochondria in the pathogenesis of the neurodegenerative process was reported when complex I deficiency was detected in the substantia nigra and platelet mitochondria of patients with PD. Furthermore, strong evidences were found for ETC deficiencies: complex I and cytochrome *c* oxidase (complex IV, COX) in AD and complexes II and III in HD [47, 48].

PD is the earliest neurodegenerative disease associated with mitochondrial dysfunction. As a potent neurotoxin, 6-OHDA causes the degeneration of dopaminergic neurons. Thus, it has been used as a selective catecholaminergic neurotoxin to establish cell and animal models of PD. In the study of Tian et al. [44], after SH-SY5Y cells were treated with Sal B (0.1-10 M), a decrease of the mitochondrial membrane potential and an increase of intracellular Ca^2+^ induced by 6-OHDA were significantly attenuated in a concentration-dependent manner.

Mitochondrial dysfunction is also a featured pathology underlying synaptic injury and neuronal stress in AD. In the study of He et al. [35], the protective effect of Sal B against A*β*-induced mitochondrial abnormalities in primary neurons of C57BL/6J mice was determined. Results showed that Sal B (50-400 *μ*M) substantially suppressed excessive mitochondrial superoxide generation in A*β*-insulted neurons. Moreover, Sal B has demonstrated strong protection on mitochondrial bioenergetics against A*β* toxicity evidenced by preserved mitochondrial membrane potential and ATP production, as well as rescued enzymatic activities of cytochrome *c* oxidase and F_1_F_O_ ATP synthase. In addition, A*β*-induced axonal mitochondrial fragmentation and increased dynamin-like protein 1 phosphorylation at Ser 616 were substantially mitigated by Sal B.

## 7. Neurogenesis Promotion of Sal B

The adult mammalian's brain maintains the ability for neurogenesis, which mainly focuses in the subventricular zone (SVZ) and the subgranular zone (SGZ) [49]. In SVZ, neural stem cells (NSCs) are relatively quiescent radial glia-like cells, which divide infrequently to produce fast-dividing precursor cells (NPCs). NPCs act as intermediate cell types, which generate neuroblasts. The majority of neuroblasts move along the rostral migratory stream to the olfactory bulb, where they largely differentiate into GABAergic neurons. In SGZ, primary NSCs also have low proliferative activity and classical astrocytic features. These cells could give rise to two types of transit-amplifying neuroblasts and then migrate into the dentate granule cell layer of the hippocampus, wherein they differentiate into glutamatergic neurons and integrate into the local circuitry [50]. There are multiple modulators that affect the formation of newborn neurons, such as neurotrophic factors, cytokines, chemokines, epigenetic factors, and signaling pathways. Each modulator drives NSC proliferation, differentiation, migration, and survival in different ways [51]. Severe brain injury and neurodegenerative diseases often initiate neurogenesis in the brain, and increasing evidences suggested that the promotion of neurogenesis could improve the decline of spatial memory induced by AD [52].

Guo et al. found that Sal B (20 *μ*g/mL, 40 *μ*g/mL) can promote the proliferation of NSCs derived from the cortex of fetal mice, increase the number of NSCs and cultured neurospheres, and promote NSCs to differentiate into neurons. And it may be related to the DNA synthesis at the S and G2 phases [53]. Zhuang et al. have screened 45 natural compounds derived from Chinese herbal medicines for the neuroproliferation-inducing activity in neural stem/precursor cells (NSPCs). Results showed that Sal B (20 *μ*M) significantly increased the survival rate of NSCs and maintained the self-renewal ability of NSCs by upregulating the mRNA expression of Nestin and Notch-1, a mechanism which may be related to the PI3K/Akt pathway [54]. Zhong et al. studied the role of Sal B in rats with middle cerebral artery occlusion which was induced by the intraperitoneal injection of BrdU-labeled SVZ cells. Immunofluorescence results showed that ischemia/reperfusion injury increased BrdU-positive cell number compared with the control group, while Sal B (1 mg/kg, 10 mg/kg) could significantly promote neurogenesis [55]. Zhang et al. labeled BrdU on bone marrow-derived neural stem cells (BM-NSCs) cultured in vitro and investigated the effect of different concentrations of Sal B (5 mg/mL, 20 mg/mL, and 40 mg/mL) on their proliferation. The results showed that Sal B can promote nestin-positive BM-NSCs to mainly differentiate into NF-M-positive neurons and NG2-positive oligodendrocytes, while few of them differentiated into GFAP-positive astrocytes [56].

The treatment targets, cell lines/animal models, doses, and the possible mechanism of Sal B on neurodegenerative diseases are summarized in Table 1.

## 8. Conclusion

Previous studies have demonstrated that Sal B could exert a therapeutic effect on neurodegenerative diseases, the mechanism of which involves inhibiting A*β* aggregation and fiber formation, decreasing A*β* neurotoxicity, decreasing tau protein hyperphosphorylation, preventing neuroinflammation and oxidative damage, inhibiting apoptosis, restoring mitochondrial dysfunction, and promoting neurogenesis. All the results mentioned above indicate that Sal B has the potential to become a novel and prospective drug for the treatment of neurodegenerative diseases. However, we have to realize that the beneficial effect of Sal B on AD, PD, and HD still needs to be confirmed in larger animals or even in humans before they are applied in clinical settings, and the optimal dosage of Sal B also needs to be determined in further studies.

With the ongoing deep research on neurodegenerative diseases, various studies have demonstrated its complexity and multifarious factors are related to its occurrence and development, including genetic factors, dysregulation of calcium homeostasis in nerve cells, and abnormal energy metabolism. In addition, a series of intertwined physiological and biochemical reactions happen during the pathological process, which makes the discovery of potential drug targets very difficult. Thus, employing a comprehensive and multiple approach to facilitate and push our researches is necessary; for example, conducting investigations on knocking out the potential gene in animals and modifying the molecular structure of Sal B to improve activity. In a word, for the sake of a clear illumination of the underlying mechanism of Sal B against with neurodegenerative diseases, a lot of work needs to be done in the future.

## Figures and Tables

**Table 1:** Treatment targets, cell lines/animal models, doses, and the possible mechanism of Sal B on neurodegenerative diseases.

Treatment targets	Cell lines/animal models	Doses	Possible mechanism	References
A*β* aggregation and fiber formation	PC12 cells	10 nmol/L	Inhibit fibrogenesis of A*β* and damage the formed A*β* fiber; reduce the accumulation of reactive oxygen; inhibit the aggregation and formation of the A*β*_1-40_ fiber	[20]
	PC12 cells	1-100 *μ*M	Inhibit A*β*_40_ fiber formation (IC_50_: 1.54-5.37 *μ*M); damage the formed A*β*_40_ fiber (IC_50_: 5.00-5.19 *μ*M); reduce the accumulation of reactive oxygen induced by A*β*_25-35_ aggregation	[21]
	SH-SY5Y-APPsw cells	25 *μ*M, 50 *μ*M, 100 *μ*M	Decrease the protein expressions of sAPP*β* and *β*-site APP-cleaving enzyme 1; increase the levels of sAPP*α* and antidisintegrin and metalloprotease 10	[22]

A*β* neurotoxicity	PC12 cells	10 nmol/L, 100 nmol/L	Reduce toxicity of A*β*_40_	[24]
	Injury model on primary rat cortical neuron using 5 *μ*mol/L A*β*_40_	0.01 *μ*g/L, 0.10 *μ*g/L, 1.00 *μ*g/L	Increase cell viability; dose-dependently reduce the release rate of LDH and NO; alleviate the toxicity of A*β*_40_ on the cortical neuron	[25]

Tau protein hyperphosphorylation	SH-SY5Y cells after incubation with 50 nmol/L OA for 36 h	0.072 *μ*g/mL, 0.72 *μ*g/mL	Increase cell activity; reduce the proportion of apoptosis	[28]
	SH-SY5Y-APPsw cells	50 *μ*M, 100 *μ*M	Increase phosphorylation at Ser9 of GSK-3*β*	[22]

Anti-inflammatory and antioxidant effects	Rats were intraventricularly injected with A*β*_25-35_	10 mg/kg	Decrease the number of microglia (OX-42 positive) and astrocytes (GFAP positive); decrease the mRNA expression of iNOS and COX-2	[33]
	Mice model of traumatic brain injury	25 mg/kg	Reduce expressions of proinflammatory cytokines TNF-*α* and IL-1*β*; increase the expression of anti-inflammatory factors IL-10 and TGF-*β*1	[34]
	C57BL/6J mice was exposed to 500 nM A*β*	50-400 *μ*M	Alleviated intraneuronal glutathione (GSH) and lipid oxidation	[35]
	Microglia-neuron coculture system	50 *μ*M for anti-inflammatoryeffect; 0.5, 5, and 50 *μ*M for antioxidant effect	Decrease the production of NO, TNF-*α*, and IL-1*β*; decrease the mRNA expressions of iNOS, TNF-*α*, and IL-1*β*; dose-dependently reduce the amount of production of ROS in microglia cells; inhibit the activation of transcription factor NF-*κ*B in microglia	[36]

Apoptosis	PC12 cells treated with A*β*_25-35_	10 nmol/L, 100 nmol/L, 1 *μ*mol/L	Reduce the proportion of apoptosis	[20, 41]
	SH-SY5Y cells exposed to MPP+ (500 *μ*Μ)	50 *μ*M, 100 *μ*M	Stabilization of mitochondrial membrane potential; decrease of apoptotic cell number and ROS production; inhibit the release of Cyt *c* and the activation of caspase-3; reduce bax expression to the normal level; reduce the ratio of bax/bcl-2	[43]
	PD models of SH-SY5Y exposed to 6-OHDA (100 *μ*M) for 12 h	0.1 *μ*M, 1 *μ*M, 10 *μ*M	Dose-dependently reduce the activity of caspase-3; inhibit the increase in bax and the decrease of bcl-2; increase the proportion of bcl-2/bax	[44]

Mitochondrial dysfunction	SH-SY5Y cells exposed to 6-OHDA	0.1-10 M	Attenuate the decrease of mitochondrial membrane potential and increase of intracellular Ca^2+^ in a concentration-dependent manner	[44]
	Primary neurons of C57BL/6J mice exposed to A*β*	50-400 *μ*M	Suppress excess mitochondrial superoxide generation; protect mitochondrial bioenergetics against A*β* toxicity by preserving mitochondrial membrane potential and ATP production; rescue enzymatic activities of cytochrome *c* oxidase and F_1_F_O_ ATP synthase; mitigate axonal mitochondrial fragmentation and increased dynamin-like protein 1 phosphorylation at Ser 616	[35]

Neurogenesis	NSCs derived from the cortex of fetal mice	20 *μ*g/mL, 40 *μ*g/mL	Promote the proliferation of NSCs; increase the number of NSCs and cultured neurospheres; promote NSCs to differentiate into neurons	[53]
	Neural stem/precursor cells (NSPCs)	20 *μ*M	Increase the survival rate of NSCs; maintain the self-renewal ability of NSCs by upregulating the mRNA expression of Nestin and Notch-1	[54]
	Rats with middle cerebral artery occlusion	1 mg/kg, 10 mg/kg	Increase the number of SVZ cells	[55]
	Bone marrow-derived neural stem cells (BM-NSCs)	5 mg/mL, 20 mg/mL, 40 mg/mL	Promote nestin-positive BM-NSCs to mainly differentiate into NF-M-positive neurons and NG2-positive oligodendrocytes; few of them differentiated into GFAP-positive astrocytes	[56]
